# Tracheotomy for Abscess in the Root of the Tongue, Resulting Successfully

**Published:** 1850-11

**Authors:** Daniel Brainard

**Affiliations:** Prof. of Surgery in Rush Medical College; Chicago


### ARTICLE VIII.

*Tracheotomy for Abscess in the root of the tongue, resulting successfully.*—By DANIEL BRAINARD, M.D., Prof. of Surgery in Rush Medical College.

On the 30th June, 1850, I was invited by Dr. Bassett, of this city, late in the evening, to see a child about one year old laboring under the most urgent symptoms of suffocation. There was a swelling beneath the left angle of the jaw extending forward beneath the chin; the tongue was swollen at its base, and the tonsils were much enlarged. The swelling had existed and gradually increased for about a week. On examining the tumor beneath the angle of the jaw I thought fluctuation was perceptible, and, passing a bistoury deeply into it, had the satisfaction of finding it followed by a copious discharge of pus.

Not doubting that this would be followed by prompt relief I made preparation to leave the house, when I noticed that the child's breathing became more difficult and instant death was threatened, when I proceeded, as rapidly as possible, to open the trachea. This gave immediate relief. On passing a probe into the opening of the abscess, it passed above the os hyoides and in front of the epiglottis nearly to the opposite side. This opening was kept pervious by the introduction of a piece of bougie; breathing continued partially through the artificial opening for about a month, when it closed, the child being quite well. Abscesses in the base of the tongue and at the sides of and behind the pharynx, are often fatal. In an urgent case called idiopathic glossitis, of several days' standing, I plunged a bistoury in the median line, midway between the chin and os hyoides, upward and backward, about two inches; inserting a tent, a free discharge of pus soon took place and the patient was relieved.

After the operation for Tracheotomy I find the best way of preventing the opening from closing is by ligatures passed through the tissues on each side and tied behind the neck.

Chicago, Oct. 1, 1850.

